# Association between *CRP* genetic diversity and bipolar disorder comorbid complications

**DOI:** 10.1186/s40345-017-0109-1

**Published:** 2018-01-20

**Authors:** Wahid Boukouaci, José Oliveira, Bruno Etain, Meriem Bennabi, Christina Mariaselvam, Nora Hamdani, Céline Manier, Djaouida Bengoufa, Frank Bellivier, Chantal Henry, Jean-Pierre Kahn, Dominique Charron, Rajagopal Krishnamoorthy, Marion Leboyer, Ryad Tamouza

**Affiliations:** 1 INSERM, U1160, Hôpital Saint Louis, 75010 Paris, France; 2 Fondation FondaMental, 94000 Créteil, France; 3 AP-HP, Département de psychiatrie et de medicine addictologique, Hôpital Fernand Widal, Paris, France; 4 INSERM, UMR-S1144–VariaPsy, Hôpital Fernand Widal, 75010 Paris, France; 5 Université Paris Diderot, Sorbonne Paris-Cité, 75013 Paris, France; 6 INSERM, U955, Psychiatrie Translationnelle, 94000 Créteil, France; 7 Faculté de Médecine, Université Paris-Est, 94000 Créteil, France; 8 AP-HP, DHU PePSY, Pôle de Psychiatrie, Hôpitaux Universitaires Henri Mondor, 94000 Créteil, France; 9 Laboratoire Jean Dausset and LabEx Transplantex, Hôpital Saint Louis, 75010 Paris, France; 10 Service de Psychiatrie et Psychologie Clinique, CHU de Nancy, Hôpitaux de Brabois, 54500 Vandoeuvre Les Nancy, France

**Keywords:** Bipolar disorder, CRP, Polymorphisms, Early-onset bipolar disorder, Autoimmune thyroiditis, Rapid cycling

## Abstract

**Background:**

Chronic low-grade inflammation is believed to contribute, at least in a subset of patients, to the development of bipolar disorder (BD). In this context, the most investigated biological marker is the acute phase response molecule, C-reactive protein (CRP). While the genetic diversity of *CRP* was amply studied in various pathological settings, little is known in BD.

**Methods:**

568 BD patients along with 163 healthy controls (HC) were genotyped for the following single-nucleotide polymorphisms (SNPs) on the *CRP* gene: intron *rs*1417938 (+ 29) T/A, 3′-UTR *rs*1130864 (+ 1444) G/A, and downstream *rs*1205 (+ 1846) (C/T). The statistical analysis was performed using Chi-square testing and consisted of comparisons of allele/genotype frequencies between patients and controls and within patient sub-groups according to BD clinical phenotypes and the presence of thyroid disorders.

**Results:**

We found that the frequencies of the studied SNPs were similar in BD and HC groups. However, the *CRP rs*1130864 A allele carrier state was significantly more frequent: (i) in BD patients with thyroid disorders than in those without (*pc* = 0.046), especially among females (*pc* = 0.01) and independently of lithium treatment, (ii) in BD patients with rapid cycling than in those without (*pc* = 0.004).

**Conclusions:**

Overall, our findings suggest the possibility that *CRP* genetic diversity may contribute to the development of auto-immune comorbid disorders and rapid cycling, both proxy of BD severity. Such findings, if replicated, may allow to predict complex clinical presentations of the disease, a possible step towards precision medicine in psychiatry.

## Background

There is ample evidence for a dysregulated immune/inflammatory component in major psychiatric disorders such as bipolar disorder (BD) (Berk et al. 2013; Leboyer et al. 2016) and schizophrenia (Kirkpatrick and Miller 2013). Such immune dysfunction is reflected, at least in sub-groups of patients, by an underlying chronic low-grade inflammation characterized by increased circulating levels of pro-inflammatory molecules along with an elevated incidence of comorbid inflammatory medical conditions viz cardiovascular/metabolic and autoimmune disorders (Benros et al. 2014; Rosenblat and McIntyre 2015).

Among the markers of inflammation, elevated levels of C-reactive protein (CRP), a component of the innate immune system and an acute phase reactant, are a consistent finding in these disorders (Dickerson et al. 2007; Ford and Erlinger 2004; Huang and Lin 2007; Liukkonen et al. 2006). We recently extended this notion through a meta-analysis showing that CRP levels are elevated in patients with BD, particularly during manic episodes and euthymic phases (Dargél et al. 2015). Interestingly, CRP level is also an independent risk factor for cardiovascular/metabolic and autoimmune disorders including autoimmune thyroiditis, a prominent comorbidity of BD (Chakrabarti 2011). Also striking is the observation that CRP levels decrease from pre-treatment levels in a significant proportion of BD patients under lithium monotherapy, as if lithium was also acting on inflammation either directly or indirectly (Hornig et al. 1998). However, despite the reported plausible role of lithium on immune-modulation associated with potential antioxidant effects, it is still debated if the normalized values of inflammation markers in euthymic BD patients under lithium are due to a direct effect on immune-modulation or an indirect effect due to symptom stabilization (van den Ameele et al. 2016; Data-Franco et al. 2017).

The CRP molecule is produced by the liver in response to pro-inflammatory stimuli, induced either by infection, cellular stress or tissue damage (Eklund 2009). When released in the circulation, CRP activates the classical complement pathway, a key player of various pivotal innate immune processes such as inflammation and phagocytosis (Gershov et al. 2000). Moreover, the complement system has also been shown to be involved in neurogenesis and pruning of synapses in the central nervous system providing hence another link between CRP and mental/neurological physiopathology (Pekny et al. 2007; Rahpeymai et al. 2006).

Systemic basal circulating CRP levels are genetically determined and influenced by a number of loci including *CRP* gene variants per se in various ethnic groups (Martínez-Calatrava et al. 2007; Reiner et al. 2012). In this context, a meta-analysis of genome-wide association studies of over 80,000 subjects for CRP level variability allowed to identify 18 loci among which five (that include the *CRP* gene) had been known to play a role in innate and adaptive immune response pathways (Dehghan et al. 2011). Interestingly, six other loci associated with CRP levels in that study were involved in metabolic regulation of diabetes mellitus. It has been also showed that some *CRP* variants are associated with raised levels of serum CRP while others with decreased ones (Guo et al. 2014; Kong et al. 2012; Miller et al. 2005; Ottaviani et al. 2011; Russell et al. 2004).

Given the potential influence of circulating CRP on neuropsychiatric comorbidities, neurogenesis and synaptic pruning, it is important to explore the genetics of CRP in these situations (going beyond the simple assessment of CRP level) and to relate its potential role in susceptibility to these disorders and/or to comorbid conditions. Indeed, reports on the influence of *CRP* genetic variants in mental disorders are quite limited and essentially related to depression (Bufalino et al. 2013; Halder et al. 2010).

Since immune dysfunction and auto-immunity are prominent in BD, it is of particular interest to assess the CRP genetics in its context. Towards this end, we explored here the genetic diversity of *CRP* in a large French Caucasian patient cohort affected by BD with or without comorbid presentation.

## Methods

### Sample composition

Five hundred and sixty-eight BD outpatients, followed in three French university-affiliated psychiatry departments (Paris-Créteil, Bordeaux and Nancy), were interviewed by expert psychiatrists using the French version of the Diagnostic Interview for Genetic Studies (DIGS version 3.0) (Nurnberger et al. 1994). Those satisfying the Diagnostic and Statistical Manual for Mental Disorders (DSM)-IV criteria (American Psychiatric Association 1994) for type I or II or not otherwise specified (NOS) BD were included in the present study. At inclusion, all were found to be euthymic with a Montgomery-Asberg Depression Rating Scale score and a Bech-Rafaelsen Mania Rating Scale score not exceeding five (Bech et al. 1978; Montgomery and Asberg 1979). A set of demographic/clinical variables including, presence of a thyroid disorder, age at onset (AAO) and rapid cycling (both proxy of disease complexity) were recorded (Table 1).

**Table 1:** Demographic and clinical characteristics of study subjects

	BD (*n* = 568)	HC (*n* = 163)	*p* value
	Mean ± SD	Mean ± SD	
Age at inclusion	42.4 ± 13.1	41.2 ± 11.6	NS

	BD (*n* = 568)	HC (*n* = 163)	*p* value
	*n* (%)	*n* (%)	
Gender			
Male	236 (41.5)	100 (61.3)	*p* < 0.05
Female	332 (58.5)	63 (38.7)	
Age at onset^a^			
Early-onset (< 22 years)	253 (47.5)	–	
Late-onset (≥ 22 years)	280 (52.5)	–	
Thyroiditis^a^			
No	433 (84.7)	–	
Yes	78 (15.3)	–	
Rapid cycling^a^			
No	435 (86.1)	–	
Yes	70 (13.9)	–	

*BD* bipolar disorder, *HC* healthy controls, *SD* standard deviation, *NS* non-significant

^a^Data not available for all patients

One hundred and sixty-three healthy controls (HC), assessed using the National Institute for Mental Health Family Interview for Genetic Studies and DIGS for personal and family history of psychiatric disorders, were also recruited (Maxwell 1992). Inclusion criteria were based on the absence of personal or family history (first degree) of psychiatric disorders, affective disorders or suicidal behavior. Consecutive recruitment of patients and controls (all of French descent with at least three grandparents from the mainland of France) extended between February 2006 and January 2010. The age at onset (AAO) threshold (22 years) of BD corresponding to that at which the first mood episode (depressive, manic, hypomanic or mixed) occurred was defined as previously reported (Geoffroy et al. 2013) and were categorized into two sub-groups i.e., early and late (Oliveira et al. 2014). Rapid cycling status was defined (according to DSM-IV), as the occurrence of at least four major depressive, manic, hypomanic, or mixed episodes during the previous year and thyroid disorders were objectivized by routine standard biological testing. Following institutional ethical committee approval and guidelines of the research protocol, written informed consent was obtained from all participating subjects. The present study forms part of a much larger ongoing National (French) program since 2014 and entitled “Genetic and environmental susceptibility factors to bipolar disorders” (RBM0436 and C0829). The national program had previously been approved by the French medical ethics committee (Comité de Protection des Personnes (CPP) IDRCB2008_AO1465_50 VI—Pitié Salpetrière 118-08) and carried out according to the approved guidelines.

### *CRP* genotyping

Genomic DNA was extracted from EDTA-treated peripheral blood samples or B-lymphoblastoid cell lines using the Nucleon BACC3 kit (GE HealthCare, Chalfont St Giles, UK). Three single-nucleotide polymorphisms (SNPs) on the *CRP* gene, namely intron *rs*1417938 (+ 29) T/A, 3′-UTR *rs*1130864 (+ 1444) G/A and further downstream *rs*1205 (+ 1846) (C/T) were analyzed by a TaqMan^®^ 5′-nuclease assay (Applied Biosystems^®^, Foster city, CA, USA) with allele-specific fluorogenic oligonucleotide probes. The following pre-developed TaqMan^®^ assay genotyping kits were used: C_7479322_10, C_7479332_10 and C_7479334_10.

### Statistical analysis

Comparisons of genotype and allele frequencies between patients and controls were performed using the Chi-square testing. The *p* values (two tailed) were corrected (*pc*) using the Bonferroni method and findings were considered statistically significant for *pc* < 0.05. Both odds ratio (OR) and confidence interval 95% (CI 95%) were calculated to assess the relative risk conferred by a specific allele or genotype. The demographic/clinical items viz AAO, lifetime presence of thyroid disorders (analyzed according to lithium intake or not) and rapid cycling were included in the analysis. Deviation, if any, from the Hardy–Weinberg equilibrium was analyzed using the Chi-square test. Pairwise linkage disequilibrium (LD) was ascertained using the Linkage Disequilibrium Analyzer 1.0 software (Ding et al. 2003). Association analyses were performed using SPSS for Windows, Version 16.0.

## Results

Demographic and clinical characteristics of study subjects are described in Table 1. The proportion of female gender was significantly higher in patients (58.5%) than in controls (38.7%). The observed genotype distribution satisfied the expected Hardy–Weinberg proportions for both patient and control samples and the overall frequencies were comparable to those previously published in a public database (http://www.ncbi.nlm.nih.gov/).

Both *CRP rs*1130864 and *rs*1417938 polymorphisms were in complete linkage disequilibrium (LD) in our study subjects and hence only *CRP rs1130864* and *rs1205* polymorphisms (the latter not in LD with the former) were taken into consideration in further analyses. No difference was noted in the distribution of these two SNPs between patients and controls even after gender stratification (data not shown).

Analysis of the distribution of CRP genotypes revealed that the *CRP rs*1130864 A allele carrier state (homozygous and compound heterozygous combination) was significantly more frequent in BD patients with thyroid disorders than in those without (*rs*1130864 AA + AG vs. GG others: 64.1% vs 50.1%, *p* = 0.023, *pc* = 0.046; OR = 1.78, 95% CI = 1.08–2.96), the difference relatively more pronounced among females (*rs*1130864 AA + AG vs. GG: 69.6% vs 50.4%, *p* = 0.005, *pc* = 0.01; OR = 2.25, 95% CI = 1.27–4.04 in female patients, respectively, with and without thyroiditis) (Table 2). No statistically significant results were found when stratifying patients accounting for AAO. The analysis of the potential impact of lithium intake did not reveal any difference in the genotype distribution between patients with and without thyroiditis and this was also regardless of the gender (data not shown). Similarly, we also found that the *CRP* *rs*1130864 A allele carrier state was significantly more frequent in BD patients with rapid cycling than in those without (*rs*1130864 AA + AG vs. GG others: 68.6% vs 49.0%, *p* = 0.002, *pc* = 0.004; OR = 2.27, 95% CI = 1.33–3.95, respectively, in patients with and without rapid cycling) (Table 2).

**Table 2:** *CRP* rs1130864 genotype distribution in bipolar disorder patients with and without thyroid disorders

	*CRP rs*1130864		*p* value	*pc*	OR (CI 95%)
	GG (*n*, %)	AG + AA (*n*, %)			
Thyroiditis					
Yes	28 (35.9)	38 (48.7) + 12 (15.4)	0.023	0.046	1.78 (1.08–2.96)
No	216 (49.9)	178 (41.1) + 39 (9.0)			
Female patients					
Yes	21 (30.4)	36 (52.2) + 12 (17.4)	0.005	0.01	2.25 (1.27–4.04)
No	112 (49.6)	94 (41.6) + 20 (8.8)			
Early-onset BD					
Yes	11 (34.4)	16 (50.0) + 5 (15.6)	NS	NS	1.89 (0.87–4.25)
No	104 (49.8)	89 (42.6) + 16 (7.7)			
Rapid cycling					
Yes	22 (31.4)	34 (48.6) + 14 (20.0)	0.002	0.004	2.27 (1.33–3.95)
No	222 (51.0)	176 (40.5) + 37 (8.5)			

*CRP* C-reactive protein, *OR* odds ratio, *CI* confidence interval

## Discussion

BD is now clearly considered as being a multisystem disorder given the elevated burden of medical comorbidities, including cardiovascular disorders, diabetes mellitus, obesity and auto-immune disorders, the majority of them likely related to the often observed immune dysfunction (Leboyer et al. 2012). According to the dual implication of CRP molecules in both acute and chronic inflammatory processes (Black et al. 2004) and given the known genetic control governing their production, we investigated here the distribution of three *CRP* SNPs namely *rs*1417938, *rs*1130864, and *rs*1205, all having functional relevance in terms of CRP expression as suggested by previous studies (Halder et al. 2010; Ottaviani et al. 2011; Zacho et al. 2008; Zakharyan et al. 2010).

The observed absence of association in the whole sample studied here might be explained either by the relatively small sample size and/or by the well-known clinical heterogeneity characterizing BD. Merely, as BD could semantically be compared to an “umbrella” covering several disorders governed by specific mechanisms, the genetic association observed herein with sub-forms of the disorder may possibly reflect different operating immunological processes. Nevertheless, lack of association in case–control analyses is in agreement with previous studies exploring the genetics of CRP production in BD and other psychiatric diagnoses using much larger sample sizes (Avramopoulos et al. 2015; Prins et al. 2016).

When analyzing patients with thyroid disorders, we found that BD male and female patients differed in terms of SNPs distribution, a notion already attributed to differences in hormonal, behavioral, and lifestyle factors in the context of the disorder (Hillegers et al. 2007; Hornig et al. 1998; Kupka et al. 2002). In particular, the prevalence of thyroid-related disorders, namely the autoimmune ones, is known to be much higher in females than in males (Bauer et al. 2014; Özerdem et al. 2014). This may explain why we observed that the 3′-UTR *CRP rs*1130864 A minor allele carrier state is associated with autoimmune thyroiditis stringently in female patients.

In addition, we also found that such association is independent of lithium treatment, a finding in agreement with previous reports showing that autoimmune thyroiditis is a comorbid condition occurring independently of lithium uptake (Kupka et al. 2002; Padmos et al. 2004). Since CRP levels tend to decrease in BD patients on lithium monotherapy, lithium treatment would be expected to mask the elevated levels of CRP in thyroid disorders, if the analysis were to be based solely on the serum CRP levels (Hornig et al. 1998). We also show that the 3′-UTR *CRP rs*1130864 A allele carrier state is more prevalent among patients with rapid cycling, which is relevant since thyroid disorders and auto-immune abnormalities are associated with refractory forms of BD, in particular, rapid cycling (Chakrabarti 2011; Padmos et al. 2004), even though these two phenotypes were not correlated in our cohort.

Although our results warrant replication, it is of interest to hypothesize that a subgroup of BD patients is associated with *CRP* gene variants, namely female patients having thyroid disorders and/or rapid cycling suggesting that abnormal inflammatory processes may be a link between these apparently distinct clinical features.

## Conclusions

To confirm the role of CRP, our findings need to be replicated in a larger sample along with available levels of circulating serum levels of CRP. Within the field of “immunopsychiatry” attempts to identify meaningful sub-forms of BD, relevant to specific underlying mechanisms may lead to specific innovative therapeutic strategies.

## Abbreviations

BD: bipolar disorder
CRP: C-reactive protein
DIGS: Diagnostic Interview for Genetic Studies
DSM-IV: Diagnostic and Statistical Manual for Mental Disorders (DSM)-IV
NOS: not otherwise specified
HC: healthy controls
SNP: single-nucleotide polymorphism
LD: linkage disequilibrium
3′-UTR: 3′ untranslated region
